# A clinical and molecular characterization of a Pakistani family with multicentric osteolysis, nodulosis and arthropathy (MONA) syndrome

**DOI:** 10.1016/j.bonr.2024.101789

**Published:** 2024-07-15

**Authors:** Safeer Ahmad, Mari Muurinen, Petra Loid, Muhammad Zeeshan Ali, Muhammad Muzammal, Sana Fatima, Jabbar Khan, Muzammil Ahmad Khan, Outi Mäkitie

**Affiliations:** a Gomal Center of Biochemistry and Biotechnology, Gomal University, Dera Ismail Khan, Pakistan; b Research Program for Clinical and Molecular Metabolism, Faculty of Medicine, University of Helsinki, Helsinki, Finland; c Children's Hospital, University of Helsinki and Helsinki University Hospital, Helsinki, Finland; d Folkhälsan Research Center, Genetics Research Program, Helsinki, Finland; e Institute of Biological Sciences, Gomal University, Dera Ismail Khan, Pakistan

**Keywords:** MONA, Osteolysis, MMP2, Pakistani family, Molecular & *in silico* analysis

## Abstract

Multicentric osteolysis nodulosis and arthropathy (MONA) is a rare skeletal dysplasia characterized primarily by progressive osteolysis, particularly affecting the carpal and tarsal bones, accompanied by osteoporosis. In addition, it features subcutaneous nodules on the palms and soles, along with the progressive onset of arthropathy, encompassing joint contractures, pain, swelling and stiffness. It is caused by a deficiency of the Matrix Metalloproteinase-2 (MMP2). In the current study we present a comprehensive clinical, radiological, genetic and *in silico* analysis of MONA in a consanguineous Pakistani family. Clinical and radiological examinations of the three severely affected siblings demonstrated a progressive MONA syndrome with phenotypic variability. The patients presented unusual facial appearance, thickened skin, severe short stature, short hands and feet. Radiographs revealed extensive bone deformities affecting upper and lower arms, legs, vertebrae and hip. Genetic analysis revealed a homozygous missense variant [c.539 A > T p.(Asp180Val)] in the *MMP2* gene. *In silico* findings suggested a mutant MMP2 protein with a decreased stability and an altered pattern of interactions. Our findings add to the existing literature on the skeletal phenotype of MONA syndrome, including the specific clinical and radiological patterns observed. Moreover, the study will aid in genetic counseling and accurate diagnosis of families affected by the same disorder within the Pakistani population.

## Highlights

• We report on a Pakistani family affected by severe MONA syndrome.
• Clinico-genetic analysis together with *in silico* methods confirmed the disease-causing effect of the identified homozygous *MMP2* variant.
• Our findings facilitate accurate diagnosis and genetic counseling in MONA syndrome.

## 1 Introduction

The inherited osteolysis conditions are a group of rare disorders characterized by the destruction and resorption of bones, resulting in skeletal abnormalities and impaired functioning (Al Aqeel et al., 2000). Multicentric Osteolysis, Nodulosis, and Arthropathy (MONA) syndrome is a broad term used to describe very rare subtypes of skeletal dysplasias, primarily osteolytic in nature with specific involvement of carpal and tarsal bones. Additionally, the spectrum involves Winchester syndrome (Winchester et al., 1969) also referred to as Torg osteolysis syndrome, the nodulosis arthropathy osteolysis (NAO) syndrome, and Torg-Winchester syndrome. MONA syndrome follows an autosomal recessive inheritance pattern.

The typical skeletal features of MONA syndrome include progressive hand and foot deformities accompanied by carpal and tarsal bone osteolysis and osteopenia. Subcutaneous nodules can appear with deformities of the hands and feet, and patients can suffer from an arthropathy-like condition with pain, stiffness, and swelling. One characteristic feature of the observed deformities in MONA is the flexion deformity of the proximal interphalangeal joints (known as camptodactyly) in the hands. Over time, contractures may progress and involve larger joints, leading to increased disability. This pattern of joint involvement typically occurs in a similar manner in the feet as well. In addition to skeleton, facial dysmorphism, oral (Bhavani et al., 2016; De Vos et al., 2019; Ekbote et al., 2014; Jeong et al., 2010; Temtamy et al., 2012; Tuysuz et al., 2009; Winchester et al., 1969), ophthalmological, and cardiac manifestations (Bhavani et al., 2016; Castberg et al., 2013; De Vos et al., 2019; Ekbote et al., 2014; Gok et al., 2010; Jeong et al., 2010; Kröger et al., 2019; Temtamy et al., 2012; Tuysuz et al., 2009; Winchester et al., 1969) have been observed.

The genetic etiology of MONA syndrome involves inactivating mutations in the Matrix Metallopeptidase 2 (*MMP2*) gene, which encodes the enzyme matrix metalloproteinase-2, also known as gelatinase A. This enzyme is responsible for breaking down certain components of the extracellular matrix and also plays a crucial role in cellular attachment, differentiation, growth, and apoptosis (Mosig et al., 2007). The MMP2 protein is composed of several functional domains, which include a signal peptide, propeptide domain, catalytic domain, and hemopexin domain (Morgunova et al., 1999). The propeptide of MMP2 contains a cysteine motif (Pro100-Arg-CysGly-Asn-Pro-Asp106), which binds to the catalytic zinc ion and helps to maintain the enzyme in an inactive, or latent, state (Tallant et al., 2010). Among the functional domains of MMP2, the catalytic domain is the most highly conserved and contains three inserts of fibronectin type 2 repeats. In contrast, the hemopexin domain serves as the binding site for tissue inhibitors of metalloproteinases (TIMPs) (Morgunova et al., 2002). Recent studies have shown that *Mmp2* null mice exhibit an attenuated form of MONA syndrome, characterized by abnormal development of craniofacial structures, arthropathy, and decreased bone mineral density (Mosig et al., 2007). To date, 27 different *MMP2* mutations associated with MONA, Torg-Winchester syndrome (TWS), and Torg syndrome (TS) have been reported (Azzollini et al., 2014; Bader-Meunier et al., 2016; Bhavani et al., 2016; Castberg et al., 2013; Ekbote et al., 2014; Elsebaie et al., 2021; Gok et al., 2010; Imam et al., 2023; Ishaq et al., 2023; Jeong et al., 2010; Kröger et al., 2019; Mandragos et al., 2021; Martignetti et al., 2001; Mosig et al., 2007; Phadke et al., 2007; Posey et al., 2017; Rouzier et al., 2006; Shakiba and Alaei, 2023; Temtamy et al., 2012; Tuysuz et al., 2009; Zankl et al., 2005; Zankl et al., 2007). Further identification and characterization of individuals with mutations in the *MMP2* gene could potentially yield valuable insights into the physiological function of MMP2.

Here in, we present a consanguineous Pakistani family in which three siblings were affected by progressive but clinically variable MONA syndrome. Molecular analysis identified a known homozygous pathogenic missense variant in the *MMP2* gene. *In silico* findings suggested the mutant MMP2 protein to have decreased stability and altered interaction pattern.

## 2 Materials and methods

### 2.1 Study approval and subjects

The current study was carried out according to the Declaration of Helsinki and ethically approved by the Institutional Review Board (IRB) of Gomal University, Dera Ismail Khan, Pakistan. This study presents a consanguineous Pakistani family with an autosomal recessive condition diagnosed as MONA. Information about the family history was collected and affected individuals were examined at the local governmental hospital. Blood samples were obtained from all consenting unaffected and affected individuals for genetic analysis. Written informed consent from all study participants were obtained for the molecular experimentation and publication of the data.

### 2.2 DNA extraction and quantification

Genomic DNA from the available blood samples was extracted according to standard protocols. Quantification and purity of the gDNA was measured through spectrophotometer (Nanodrop, Thermo Fisher, Europe).

### 2.3 Molecular analysis

Genomic DNA sample of a single affected member was subjected to whole-exome sequencing (WES). Exome library preparation was performed with the xGen Exome Research Panel v2 (Integrated DNA Technologies, Coralville, Iowa, USA) capture kit and sequencing was performed on a Novaseq 6000 (Illumina, San Diego, CA, USA) with average on-target coverage of 112× and 104×. Reads were aligned to GRCh37/Hg19 using Burrows-Wheeler Aligner (BWA-MEM) and variant calling was performed using Picard (v2.26.10), and Genome Analysis Toolkit (GATK) (v4.2.0.0). Variants were annotated using ANNOVAR (July 2017). Based on a recessive inheritance pattern observed in the pedigree, manual filtration criterion was set to exonic and splice-site homozygous and compound heterozygous variants with minor allele frequency (MAF) ≤ 0.005 in the genome aggregation database (gnomAD) [https://gnomad.broadinstitute.org/] (Lek et al., 2016) and a scaled C-score of ≥ 20 in the combined annotation dependent depletion (CADD) database [https://cadd.gs.washington.edu/] (Rentzsch et al., 2021). The databases, InterVar [https://wintervar.wglab.org/] (Li and Wang, 2017), VarSome [https://varsome.com/] (Kopanos et al., 2019) and ClinVar [https://www.ncbi.nlm.nih.gov/clinvar/] were used to evaluate the clinical significance of identified candidate variant (as per ACMG guidelines) (Richards et al., 2015). Sanger DNA sequencing was performed to confirm the segregation of the candidate variant with disease within the family. Primer3web (version: 4.1.0) [https://primer3.ut.ee/] (Untergasser et al., 2012) was used to design the primers. Di-deoxy chain termination method was used for bidirectional sequencing of the *MMP2* and the mutation analysis was performed using UGENE (version 41.0) (Okonechnikov et al., 2012).

### 2.4 *In silico* analysis

The significance of the structural and functional role of an amino acid position is a key factor in determining its rate of evolution. The conservation analysis of the *MMP2* variant was carried out using Clustal Omega [https://www.ebi.ac.uk/Tools/msa/clustalo/] (Madeira et al., 2022). A web based tool, MUpro [http://mupro.proteomics.ics.uci.edu] (Cheng et al., 2006) was utilized to predict the stability of the mutant MMP2 protein. Each amino acid has its own specific size, charge, and hydrophobicity-value. We used another web based service “HOPE” [https://www3.cmbi.umcn.nl/hope/] (Venselaar et al., 2010) to *analyze the structural effect of the mutant MMP2 residue*. Finally, we performed interaction analysis between wildtype and mutant MMP2 protein with their close interactors using STRING (prediction of close interactor) [https://string-db.org/] (Szklarczyk et al., 2019), clusPro 2.0 (interaction analysis) [https://cluspro.bu.edu/login.php] (Vajda et al., 2017) and Chimera 1.13.1(Pettersen et al., 2004) and LigPlot+ (Version 2.1) (molecular visualization) (Laskowski and Swindells, 2011).

## 3 Results

### 3.1 Clinical description

**Fig. 1:**
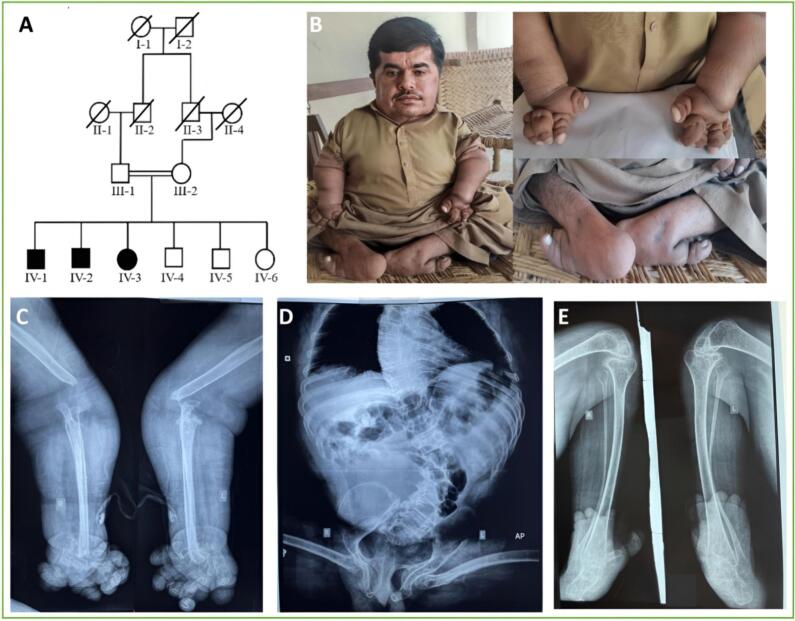
(A) Pedigree showing autosomal recessive inheritance. (B) Images of an affected member (IV-1) displaying multiple osteolysis syndromic features. (C–E) Radiographs of the patient (IV-1) at the age of 32 years. (C) Missing distal humerus, deformed distal ends of ulna and radius, and severe destruction of carpal, metacarpal and phalangeal bones. (D) Severe scoliosis of the spine, distorted pelvis, deformed and dislocated femoral heads and abnormal trabecular pattern. (E) Very thin and distorted fibula, bowed tibia, severe destruction of the ankle structures, tapering and loss of metatarsals, loss of phalangeal bones.

The recruited consanguineous Pakistani family presented a recessive condition (Fig. 1A) of progressive multiple osteolysis syndrome. Detailed clinical and radiographic examinations of each affected member are given below and summarized in Table 1.

**Table 1:** Clinical details of the patients with MONA condition.

Patient ID	IV-1	IV-2	IV-3
Gender	Male	Male	Female
Height (cm)	122	95	88
Age at onset (years)	2.5	6	3
Current age (years)	32	26	22
Coarse face	+	+	+
Bulbous nose	+	+	+
Gum hypertrophy	−	−	−
Deformed hands and feet	+	+	+
Swelling	+	+	+
Cardiac defects	−	−	−
Hyperpigmentation of skin	+	+	+
Hirsutism	−	−	+
Wide metacarpals/metatarsals	+	+	+
Osteolysis of carpal/tarsal bones	+	+	+
Osteoporosis	+	+	+
Spine defect	+	+	N/A
Ophthalmic impairment	−	−	−

+: Present, −: Absent, N/A: Not applicable.

#### 3.1.1 Patient I

In the present family, the affected individual IV-1 was a 32-year-old man with a height of 4 ft (122 cm) and a head circumference of 23″ (58 cm). He was the eldest of six children of healthy parents who were first cousins. He had normal growth and development until the age of 2.5 years. After that, he developed progressive painful swellings and tenderness on the proximal parts of his hands and feet, and flexion deformities of the knees and hips. Physical examination revealed an unusual facial appearance, and short stature with obvious skeletal deformities. He had a large head compared to height, narrow nasal bridge, bulbous nose, full cheeks, and micrognathia (Fig. 1B). Elbows and wrists were distorted and deformed with fixed flexion of elbows. His hands were deformed with thickened skin and short fingers. Hips were adducted and flexed in a fixed position (Fig. 1B). Knees were stiff and fixed in hyperextension. Both ankles showed remarkable bending and toes were deformed (Fig. 1B). Neck and shoulder movements were normal. Dentition was normal, and there was no evidence of cleft palate or gingival hypertrophy. A computed tomography (CT) scan of the brain was also performed, and no gross bony abnormality was observed. Cognitive, ophthalmologic, and audiologic findings were normal. Radiographic analysis revealed significant skeletal anomalies consistent with osteolysis, including absent distal humerus, deformities in ulna and radius, extensive bone destruction in hands and feet, severe scoliosis, pelvic distortion, deformed femoral heads, degradation in ankle and foot structures, and abnormal trabecular pattern (Fig. 1C–E).

#### 3.1.2 Patient II

**Fig. 2:**
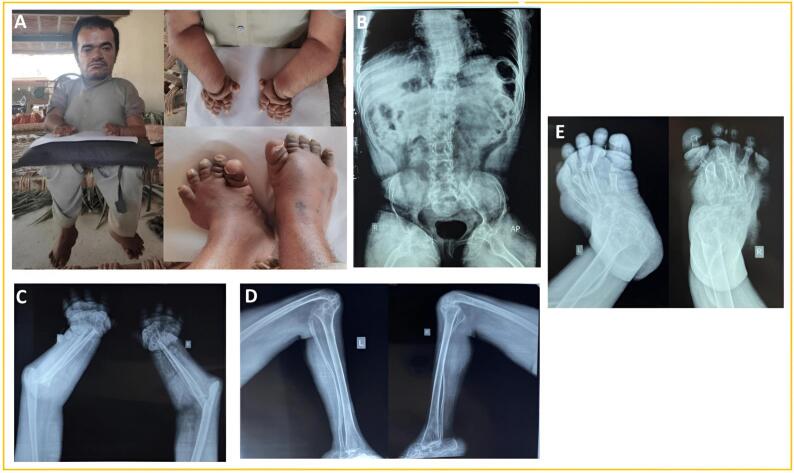
(A) Images of the affected individual IV-2 displaying the same features of MONA syndrome as his elder brother (B–E) Radiographs of the patient (IV-2) at the age of 26 years. (B) Mild scoliosis of the spine, irregularity of the femoral heads, loss of left ischiopubic ramus. (C) Severe destruction of proximal and distal ends of ulna and radius, deformed metacarpals and missing carpal and phalangeal bones. (D) Severely distorted fibula, destruction of the ankle joint. (E) Loss of talar bones, deformed metatarsal bones with distal tapering in some metatarsals, deformed and partly missing phalangeal bones.

The affected individual (IV-2) is the younger brother of the first patient. He was a 26-year-old man with a height of 3.11 ft (95 cm) and a head circumference of 23″ (58 cm). He was asymptomatic until the age of 6 years. Later, he experienced the same clinical manifestations as the elder affected brother. He had an unusual facial appearance, short stature, a large head, a narrow nasal bridge, a bulbous nose, full cheeks, micrognathia, deformed elbows and wrists, deformed hands with foreshortening of all fingers with thickened skin, adducted hips, stiff knees and deformed swollen feet with minor severity compared to the elder brother (Fig. 2A). Similarly to his brother, he also could not stand or walk and had to rely on others for his daily activities. There were no signs of cleft palate or gingival hypertrophy, and the dentition was normal. Intellectual, ophthalmologic, and audiologic findings were normal. The radiographs displayed spinal scoliosis, irregular femoral heads, and loss of the left ischiopubic ramus. Additionally, they showed severe destruction and deformities in the upper and lower limb bones, including missing carpal and phalangeal bones, distorted fibula, ankle joint destruction, and abnormalities in metatarsal and phalangeal bones (Fig. 2B–E).

#### 3.1.3 Patient III

The affected individual (IV-3) was the third affected individual of the family, the sister to IV-1 and IV-2. She was a 22-year-old female with a height of 2.9 ft (88 cm) and head circumference of 21″ (53 cm). She had normal growth and development until 3 years of age. Later, she developed similar clinical features as her brothers. She, like her brothers, was unable to stand or walk and had to rely on others for her routine tasks. There were no signs of cleft palate or gingival hypertrophy, and the dentition was normal**.** Intellectual, ophthalmologic, and audiologic findings were also normal. The parents did not agree to give permission for the images and radiographs due to cultural reasons.

### 3.2 Molecular findings

**Fig. 3:**
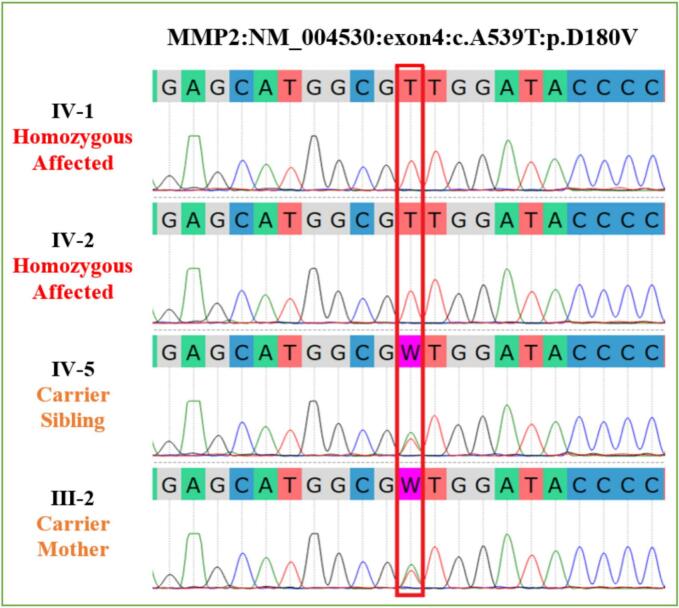
Sequence chromatogram of exon 4 of *MMP2*, demonstrating individuals **IV-1** and **IV-2** as homozygous affected and individuals **IV-5** and **III-2** as heterozygous carriers. Red box highlights the position of the substitution mutation [c.539 A > T: p.(Asp180Val)].

After carefully assessing the clinical characteristics of the affected individuals, we next carried out WES analysis to identify the genetic cause of the disease. Here, we discovered a homozygous missense variant [NM_004530:c.539 A > T:p.(Asp180Val)] in the fourth exon of the *MMP2* gene. The MAF of this allele in gnomAD was 0.000007953, with no known homozygotes and a CADD-Phred score of 26. According to gnomAD, the variant was present in the South Asian regions only with allele count 2 and absent in American, European, Ashkenazi Jews, or East Asian populations. Further, review of the South Asian population databases such as GenomeAsia100k, IndiGenomes, and South Asian Genome and Exome (SAGE) showed that the variant was absent from these databases. The VarSome database classified the variant as likely pathogenic. InterVar shows the clinical significance as VUS (variant of uncertain significance). In ClinVar, the same variant was submitted by Baylor Genetics (Jan 13, 2017) as VUS. According to ACMG classification, the variant is likely pathogenic. Sequencing analysis of the four family members confirmed the co-segregation of the missense *MMP2* variant with the disease phenotype. Family members (IV-1 & IV-2) were identified as homozygous affected and members IV-5 (unaffected brother) and III-2 (unaffected mother) as heterozygous carriers (Fig. 3).

### 3.3 *In silico* findings

**Fig. 4:**
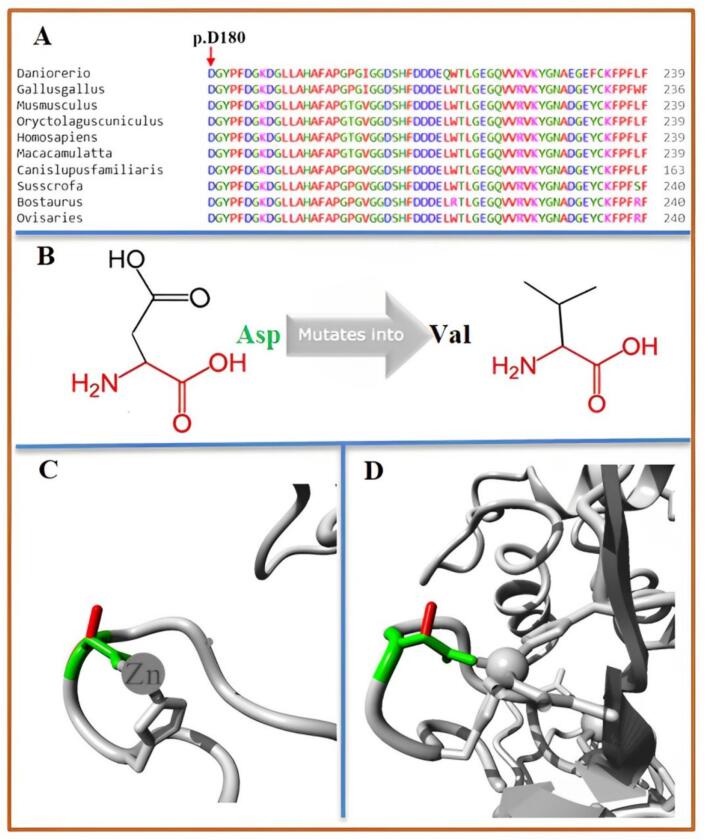
(A) Multiple sequence alignment through Clustal Omega revealed high conservation of Asp180 (MMP2) across several species. (B) Schematic structures of the wild-type amino acid (left) and the mutant (right) amino acid. The backbone, which is the same for each amino acid, is colored red. The side chain, unique for each amino acid, is colored black. (C) Close-up of the mutation. The protein is colored grey, the side chains of both the wild-type and the mutant residue are shown and colored green and red respectively. (D) Close-up of the mutation (from a slightly different angle).

**Fig. 5:**
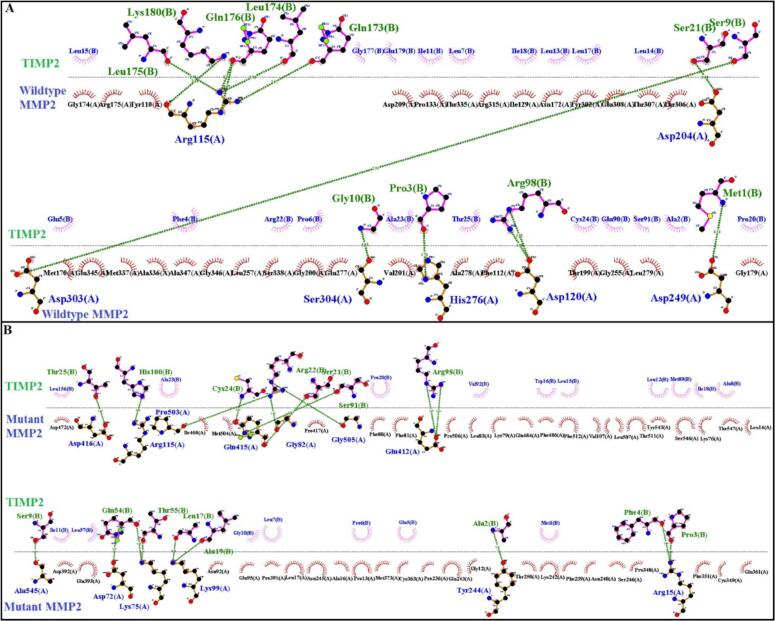
Interaction of wildtype and mutant MMP2 with the close interactor TIMP2. MMP2 residues are presented by blue color in the chain “A” while TIMP2 residues are indicated by green color in the chain “B”. Hydrogen bonds are presented by green dotted lines. (A) Seven residues (**chain A**) of the wildtype MMP2 interacting with ten residues (**chain B**) of TIMP2 *via* thirteen hydrogen bonds. (B) Thirteen residues (**chain A**) of the mutant MMP2 interacted with fifteen residues (**chain B**) of TIMP2 *via* eighteen hydrogen bonds.

Multiple sequence alignment through Clustal Omega revealed high conservation of Asp180 (MMP2) across several species (Fig. 4A). Mupro predicted the mutant MMP2 protein with decreased stability (Score: -0.09). HOPE predicted *several differences* between the wildtype (Asp180) and mutant (Val180) residues of the MMP2 protein. The mutant residue is smaller (Fig. 4B) and neutral compared to the wildtype's negative charge. Additionally, the mutant residue is more hydrophobic, potentially disrupting hydrogen bond formation and impacting the function and signal transduction within the Collagenase-like 1 region where the mutation occurs. TIMP2 was predicted as a significant interactor of MMP2 with a high confidence score. TIMP2 regulates ECM proteolysis by inhibiting MMP activity and is crucial for MMP2 activation. Docking studies revealed notable differences in interactions between normal and mutant MMP2 with TIMP2 (Fig. 5A–B), suggesting a disrupted interaction in the mutant MMP2 protein.

## 4 Discussion

MONA is a rare skeletal dysplasia mainly characterized by progressive osteolysis (especially of the carpal and tarsal bones), osteoporosis, subcutaneous nodules on the palms and soles, and progressive arthropathy (joint contractures, pain, swelling, and stiffness). Additional clinical features include coarse skin, dark skin lesions, cardiac defects, and corneal opacities. Onset typically occurs between the ages of six months and six years (range: birth to 11 years) (Mandragos et al., 2021). MONA syndromes have a significant phenotypical overlapping with Torg syndrome, Winchester syndrome and Torg-Winchester syndrome in terms of clinical manifestations, skeletal involvement and genetic etiology (Gok et al., 2010; Kröger et al., 2019; Li and Durbin, 2009; Morgunova et al., 1999; Morgunova et al., 2002; Mosig et al., 2007; Rouzier et al., 2006; Temtamy et al., 2012). Hence, they can be considered as entities belonging to the same spectrum of multisystem disorders (Tuysuz et al., 2009). Based on the Nosology and classification of genetic skeletal disorders 2023 revision, pathogenic variants in Matrix Metalloproteinase 2 (*MMP2*) and Matrix Metalloproteinase 14 (*MMP14*) have been identified to cause MONA. Both *MMP2* and *MMP14* pathogenic variants result in abnormal collagen synthesis (Bhavani et al., 2021; Elsebaie et al., 2021). To date, 53 patients from different families with 27 different biallelic pathogenic variants of the *MMP2* gene have been identified (Azzollini et al., 2014; Bader-Meunier et al., 2016; Bhavani et al., 2016; Castberg et al., 2013; Ekbote et al., 2014; Elsebaie et al., 2021; Gok et al., 2010; Imam et al., 2023; Ishaq et al., 2023; Jeong et al., 2010; Kröger et al., 2019; Mandragos et al., 2021; Martignetti et al., 2001; Mosig et al., 2007; Phadke et al., 2007; Posey et al., 2017; Rouzier et al., 2006; Shakiba and Alaei, 2023; Temtamy et al., 2012; Tuysuz et al., 2009; Zankl et al., 2005; Zankl et al., 2007). These cases have been reported in various ethnic regions including Turkey, Saudi Arabia, India, Brazil, Egypt, Italy, South America, Greece, Korea, Morocco, Algeria and Finland (Al Aqeel et al., 2000; Castberg et al., 2013). The identified *MMP2* cases with MONA, WS, TS and TWS are summarized in Table 2.

**Table 2:** Summary of previously reported subjects with *MMP2* associated osteolysis syndrome.

S.no	Study	Case (Age-years/gender)	Age of onset	Diagnosis	Cardiac defects	Genetic findings
1.	Current Study, 2024	32/Male	2.5 years	MONA	No	c.539 A > T p.Asp180Val
		25/Male	6 years	MONA	No	
		22/Female	3 years	MONA	No	
2.	(Okada et al., 2024)	47/Male	1 year	MONA	Yes	c.23_24InsG p.Gly8Glyfs*81
		45/Female	6 months	MONA	Yes	
3.	(Ishaq et al., 2023)	33/Male	17 years	MONA	No	c.1336 + 2 T > G
		31/Female	17 years	MONA	No	
		36/Male	2 years	MONA	No	c.1188C > A p.Ser396Arg
		32/Female	4 years	MONA	No	
4.	(Imam et al., 2023)	18/Female	6 years	MONA	Yes	c.732C > A p.Tyr244^a^
5.	(Shakiba and Alaei, 2023)	5.9/Female	ND	MONA	Yes	c.1648C > T p.Arg550^a^
6.	(Elsebaie et al., 2021)	6.5/Male	2 years	Mona	Yes	c.302G > A p.Arg101His
		4/Male	2 years	MONA	Yes	c.40del p.Leu14fs^a^
7.	(Mandragos et al., 2021)	14/Female	ND	MONA	No	c.529G > A p.Glu177Lys and c.1462_1464del p.Phe488del
8.	(Kröger et al., 2019)	2/Male	ND	MONA	No	c.1188C > A p.Ser396Arg
		8/Male	ND	MONA	No	
9.	(Baylor Genetics)(Posey et al., 2017)	14.7/Male	ND	Hypothyroidism, multiple joint swellings and contractures, skin anomalies and hypospadias	No	c.539 A > T p.Asp180Val and *TPO* (c.1994G > A, p.Arg665Gln)
10.	(Bader-Meunier et al., 2016)	12/Female	8 years	MONA	No	c.1188C > A p.Ser396Arg and c.1274 A > C p.Tyr425Ser
11.	(Bhavani et al., 2016)	6/Male	By birth	MONA	No	c.301C > T p.Arg101Cys
		6/Male	5.5 years	MONA	No	
		9/Female	By birth	MONA	No	c.302G > A p.Arg101His
		15/Female	3 years	MONA	ND	c.306C > A p.Cys102^a^
		11/Male	3 years	MONA	No	c.691G > T p.Glu231^a^
		4/Male	1.5 YR	MONA	Yes	c.789C > A p.Tyr263^a^
		5/Male	10 months	MONA	No	c.910_916delAGCTGCA p.Ser304Profs*115
		13/Male	11 years	MONA	ND	c.1229G > T p.Gly410Val
		ND/Male	ND	MONA	ND	
		7/Male	2 years	MONA	Yes	c.1287delG p.Asn430Thrfs*68
		7/Male	6 years	MONA	No	
		10/Female	5–6 years	MONA	No	
		13/Male	3 years	MONA	No	
12.	(Castberg et al., 2013)	5/Male	By birth	Torg syndrome with cardiac malformation	Yes	c.301C > T p.Arg101Cys
13.	(Azzollini et al., 2014)	43/Female	3–4 years	MONA	Yes	c.1228G > C p.Gly410Arg
		37/Male	3 years	MONA	Yes	
14.	(Ekbote et al., 2014)	3.5/Female	1.5 years	Torg syndrome	No	c.538G > A p.Asp180Asn
15.	(Temtamy et al., 2012)	10/Female	By birth	Torg-Winchester syndrome	Yes	c.452G > A p.Trp151^a^
		17/Female	4.5 years	Torg-Winchester syndrome	No	c.540 T > G p.Asp180Glu
		16/Male	2 years	Torg-Winchester syndrome	No	
16.	(Jeong et al., 2010)	31/Female	3 years	Torg-Winchester syndrome	No	c.1217G > A p.Gly406Asp
17.	(Gok et al., 2010)	13/Male	5 years	Torg-Winchester syndrome	No	c.658 + 2 T > C IVS4 ds T-C + 2
		10/Male	6 years	Torg-Winchester syndrome	Yes	
18.	(Tuysuz et al., 2009)	6/Male	6 months	MONA	Yes	c.1732delA p.Lys578Argfs*17
		4/Female	3 months	MONA	Yes	
19.	(Phadke et al., 2007)	9/Male	3 years	Torg–Winchester syndrome,	No	c.556G > C p.Gly186Arg
		ND	ND	Torg–Winchester syndrome,	No	
20.	(Zankl et al., 2007)	13.5/Female	8 months	MONA	No	c.302G > A p.Arg101His
21.	(Rouzier et al., 2006)	35/Female	3 years	Winchester syndrome	No	c.1488– 1490delTGG p.Val400del
		24/Female	6 months	Winchester syndrome	No	
22.	(Zankl et al., 2005)	21/Female	1–2 years	Winchester syndrome	No	c.1210G > A p.Glu404Lys
23.	(Martignetti et al., 2001)	ND/Male	8 months	MONA	No	c.732C > A p.Tyr244^a^
		ND/Female	1 years	MONA	No	
		ND/Female	ND	MONA	ND	
		ND/Female	ND	MONA	ND	
		ND/Female	ND	MONA	ND	
		ND/Female	ND	MONA	ND	
		12/Male	5 years	MONA	ND	c.302G > A p.Arg101His
		19/Male	6 years	MONA	ND	
		10/Female	6 years	MONA	ND	

Abbreviations: MONA: Multicentric osteolysis nodulosis and arthropathy, ND: Not described,

a Terminated.

Our study demonstrated a consanguineous Pakistani family with three affected individuals presenting with a progressive MONA syndrome. Genetic evaluation including WES and Sanger sequencing revealed an *MMP2* [NM_004530.4: c.539 A > T p.(Asp180Val)] variant in the family members. This variant is located in the collagenase-like 1 region in the MMP2 which is responsible for the cleavage of collagen and influences several biological processes in tissue homeostasis and disease progression. *In silico* studies predicted the mutant MMP2 protein with a decreased stability and altered interaction pattern, suggesting a disrupted MMP2 function. This variant has been previously reported by Posey et al. (2017), who retrospectively analyzed data from 7374 consecutive unrelated patients referred to a clinical diagnostic laboratory for WES (Posey et al., 2017). Of these patients, 28.2 % (2076) received a molecular diagnosis, with 4.9 % (101) having diagnoses involving multiple disease loci. Among these 101 patients, they identified two homozygous missense variants involving *TPO* [NM_000547.4: c.1994G > A, p.(Arg665Glu)] and *MMP2* [NM_004530.4: c.539 A > T, p.(Asp180Val)] in an individual with reported features of congenital hypothyroidism, multiple joint swellings and contractures, skin anomalies including brown skin pigmentation on side of abdomen and right upper arm, and hypospadias. However, detailed clinical information was not provided. Our findings demonstrated a single disease causing homozygous *MMP2* variant [NM_004530.4: c.539 A > T, p.(Asp180Val)] in the present family with characteristic features of MONA syndrome. However, hypospadia was not found in the present study patients. Hypothyroidism in the Posey et al. study is likely to be caused by the *TPO* variant.

Research conducted in the Middle East and South Asia has identified cases of MONA syndrome associated with congenital heart disease. Tuysuz et al. reported a Turkish family with an *MMP2* variant linked to congenital heart defects and skeletal abnormalities consistent with MONA syndrome (Tuysuz et al., 2009). Similarly, Tematamy et al. described Egyptian patients with Torg-Winchester syndrome, who were identified with *MMP2* gene variants (Temtamy et al., 2012). Shakiba and Alaei (2023) described a 5-year-old girl with progressive limb and facial deformities, hypertrichosis, and cardiac defects. Bhavani et al. (2016) also reported MONA syndrome cases in South Asia, two of which had associated cardiac defects (Bhavani et al., 2016; Shakiba and Alaei, 2023). In the current study, there was no clinical history of cardiac symptoms in the patients. However, none of the patients underwent cardiac ultrasound or any other cardiac test.

To conclude, MONA is a very rare and severe progressive disease. Only limited information is available on its natural progression. Even in patients with the same genetic variant, there can be significant variation in the age of onset, rate of progression and other phenotypic features, as seen in our patients. Currently, there is no specific curative treatment available for MONA, but multidisciplinary treatment may enhance the patient's quality of life. Further advances in understanding bone metabolism in this disorder may lead to new treatment possibilities. Our report adds to the existing literature on the skeletal phenotype of MONA syndrome, including the clinical and radiological features observed in the patients with the *MMP2* [NM_004530.4: c.539 A > T p.(Asp180Val)] variant. We suggest that detailed reporting of patient phenotypes, especially regarding skeletal clinical and radiographic features, can help to establish a distinct disease signature and resolve the currently unclear genotype-phenotype correlation.

## Abbreviations

MONA: multicentric osteolysis nodulosis and arthropathy
NAO: nodulosis arthropathy osteolysis syndrome
TS: torg syndrome
WS: winchester syndrome
TWS: torg-winchester syndrome
IRB: institutional review board
WES: whole-exome sequencing
MAF: minor allele frequency
gnomAD: genome aggregation database
CADD: combined annotation dependent depletion
CT: computed tomography

## Funding

This research was funded by 10.13039/501100011842Finnish National Agency for Education (EDUFI fellowship, grant number: OPH-363-2022). Open access was funded by the Helsinki University Library.

## CRediT authorship contribution statement

**Safeer Ahmad:** Writing – original draft, Investigation, Formal analysis, Data curation. **Mari Muurinen:** Writing – review & editing, Writing – original draft. **Petra Loid:** Writing – review & editing. **Muhammad Zeeshan Ali:** Formal analysis. **Muhammad Muzammal:** Methodology, Data curation. **Sana Fatima:** Methodology, Data curation. **Jabbar Khan:** Software, Data curation. **Muzammil Ahmad Khan:** Writing – review & editing, Visualization, Validation, Supervision, Project administration, Funding acquisition, Conceptualization. **Outi Mäkitie:** Writing – review & editing, Visualization, Validation, Supervision, Project administration, Funding acquisition, Conceptualization.

## Declaration of competing interest

The authors declare that they have no conflicts of interest.

## Data Availability

Data will be made available on request.
